# Effect of mometasone furoate/formoterol combination therapy on nocturnal awakenings in subjects with persistent asthma

**DOI:** 10.1186/1710-1492-7-S2-A14

**Published:** 2011-11-14

**Authors:** David Pearlman, Robert Nathan, Eli Meltzer, Hendrik Nolte, Steven Weinstein

**Affiliations:** 1 Colorado Allergy and Asthma Centers, P.C., Denver, CO, USA; 2 Asthma and Allergy Associates and Research Center, Colorado Springs, CO, USA; 3 Allergy and Asthma Medical Group and Research Center, San Diego, CA, USA; 4 Merck Research Laboratories, Kenilworth, NJ, USA; 5 Allergy & Asthma Specialists Medical Group, Huntington Beach, CA, USA

## Objective

We characterized the effect of mometasone furoate/formoterol (MF/F) treatment on nocturnal awakenings requiring rescue-medication (SABA) use in three Phase III efficacy trials (baseline=awakenings in wk prior to first dose; endpoint=awakenings across entire treatment period).

## **Methods**

Subjects were asthmatics previously using low- (n=746), medium- (n=781) or high-dose (n=728) inhaled corticosteroids ICS. Low-dose subjects were randomized to 26-wk treatment with MF/F 100/10µg, MF 100µg, F 10µg, or placebo; medium-dose subjects to 26-wk treatment with MF/F 200/10µg, MF 200µg, F 10µg, or placebo; high-dose subjects to 12-wk treatment with MF/F 400/10µg, MF/F 200/10µg, or MF 400µg. All treatments were delivered twice-daily via metered-dose inhaler.

## **Results**

Baseline awakenings ranged from 0.84–1.05 (MF/F 100/10μg study), 1.05–1.26 (MF/F 200/10μg study), and 1.33–1.61 nights/wk (MF/F 400/10μg and 200/10μg study). Nocturnal awakenings were reduced by MF/F 100/10μg=–0.42, MF 100μg=–0.21, F 10μg=–0.21, and placebo=0.14 nights/wk; changes in the other placebo-controlled study were MF/F 200/10μg=–0.56, MF 200μg=–0.35, F 10μg=+0.07 and placebo=0.00 night/wk, respectively. In both of these studies MF/F was superior to placebo (*P*<.001) and F (*P*=.035); MF was also superior to F and placebo. In the non-placebo controlled study, awakenings were reduced by –0.70, –0.70 and –0.35 nights/week by MF/F 200/10μg, MF/F 400/10μg and MF 400μg, respectively; both MF/F treatments were superior to MF (*P*≤.006).

## **Conclusions**

Both MF/F and MF significantly reduced nocturnal-awakenings compared with F and placebo. Both doses of MF/F were superior to MF in the non-placebo controlled study.

